# Development of caninized anti-CTLA-4 antibody as salvage combination therapy for anti-PD-L1 refractory tumors in dogs

**DOI:** 10.3389/fimmu.2025.1570717

**Published:** 2025-05-20

**Authors:** Naoya Maekawa, Satoru Konnai, Kei Watari, Hiroto Takeuchi, Takeshi Nakanishi, Taro Tachibana, Kenji Hosoya, Sangho Kim, Ryohei Kinoshita, Ryo Owaki, Madoka Yokokawa, Yumiko Kagawa, Satoshi Takagi, Tatsuya Deguchi, Hiroshi Ohta, Yukinari Kato, Satoshi Yamamoto, Keiichi Yamamoto, Yasuhiko Suzuki, Tomohiro Okagawa, Shiro Murata, Kazuhiko Ohashi

**Affiliations:** ^1^ Department of Advanced Pharmaceutics, Faculty of Veterinary Medicine, Hokkaido University, Sapporo, Japan; ^2^ Cancer Research Unit, One Health Research Center, Hokkaido University, Sapporo, Japan; ^3^ Department of Disease Control, Faculty of Veterinary Medicine, Hokkaido University, Sapporo, Japan; ^4^ Institute for Vaccine Research and Development (HU-IVReD), Hokkaido University, Sapporo, Japan; ^5^ Veterinary Research Unit, International Institute for Zoonosis Control, Hokkaido University, Sapporo, Japan; ^6^ Department of Chemistry and Bioengineering, Division of Science and Engineering for Materials, Chemistry and Biology, Graduate School of Engineering, Osaka Metropolitan University, Osaka, Japan; ^7^ Veterinary Teaching Hospital, Faculty of Veterinary Medicine, Hokkaido University, Sapporo, Japan; ^8^ North Lab, Sapporo, Japan; ^9^ Department of Veterinary Surgery 1, School of Veterinary Medicine, Azabu University, Sagamihara, Japan; ^10^ Companion Animal Internal Medicine, Department of Companion Animal Clinical Sciences, School of Veterinary Medicine, Rakuno Gakuen University, Ebetsu, Japan; ^11^ Department of Antibody Drug Development, Tohoku University Graduate School of Medicine, Sendai, Japan; ^12^ Fuso Pharmaceutical Industries, Ltd., Osaka, Japan; ^13^ Division of Bioresources, International Institute for Zoonosis Control, Hokkaido University, Sapporo, Japan; ^14^ Global Station for Zoonosis Control, Global Institution for Collaborative Research and Education (GI-CoRE), Hokkaido University, Sapporo, Japan; ^15^ International Affairs Office, Faculty of Veterinary Medicine, Hokkaido University, Sapporo, Japan

**Keywords:** canine tumor, immunotherapy, immune checkpoint inhibitors, cytotoxic T lymphocyte associated protein 4 (CTLA-4), programmed death ligand 1 (PD-L1)

## Abstract

Immune checkpoint inhibitors (ICIs) are widely used for cancer immunotherapy; however, the clinical efficacy of anti-PD-1/PD-L1 monotherapy is generally limited, highlighting the need to develop combination therapies. Dogs develop spontaneous tumors in immunocompetent settings, and anti-PD-1/PD-L1 antibodies exert similar clinical benefits. However, no clinically relevant anti-CTLA-4 antibody has been reported, limiting the value of canine tumors as comparative models for human ICI research. Here, canine CTLA-4 was molecularly characterized, and a caninized anti-CTLA-4 antibody (ca1C5) that blocks CTLA-4/ligand binding was developed. Treatment with ca1C5 increased cytokine production in canine immune cell cultures, and the immunostimulatory effect was enhanced when used in combination with the anti-PD-L1 antibody c4G12. As a proof-of-concept, a veterinary clinical study was conducted to demonstrate the safety and clinical efficacy of anti-CTLA-4 antibody as salvage combination therapy in dogs with advanced tumors refractory to prior c4G12 monotherapy. The combination treatment (c4G12 plus ca1C5) was well-tolerated, and evidence of antitumor activity was observed in one dog with oral malignant melanoma. Further studies are warranted to advance veterinary care for dogs and to better characterize canine ICI models for human onco-immunology research.

## Introduction

Immunotherapy has become widely available for treating various tumor types in human medicine. Immune checkpoint inhibitors (ICIs) play pivotal roles in reinvigorating exhausted T-cell responses to cancer and improving the immunosuppressive tumor microenvironment (TME) to enhance the cancer-immunity cycle (1). The immune checkpoint receptor programmed death 1 (PD-1) inhibits T-cell receptor signaling upon binding to its ligands, PD ligand 1 (PD-L1) and PD-L2 (2). PD-L1 overexpression is commonly observed in the TME of various cancer types; thus, anti-PD-1/PD-L1 antibodies can reverse T-cell suppression, leading to the induction of effective antitumor immune responses in cancer patients (3). Although patient survival can be improved, and durable response achieved with anti-PD-1/PD-L1 antibody monotherapy, the response rate is approximately 20% across many tumor types (4, 5). This suggests the existence of mechanisms that confer primary (innate) and secondary (acquired) resistance to PD-1/PD-L1 blockade (6). Cytotoxic T lymphocyte-associated protein 4 (CTLA-4) is another immune checkpoint receptor expressed on activated T cells and regulatory T cells (Tregs). CTLA-4 is a homolog of the costimulatory receptor CD28 and outcompetes it for binding to B7 ligands (CD80 and CD86), resulting in the inhibition of T-cell activation (7). Similarly, immune checkpoint blockade using anti-CTLA-4 antibodies has been shown to induce effective T cell-mediated antitumor immune responses, with clinical benefits reported in patients with melanoma (8, 9). In addition to simple receptor blockade, anti-CTLA-4 antibodies may modulate the immune response by depleting Tregs in the TME via antibody-dependent cell-mediated cytotoxicity (ADCC) (10, 11). However, the use of anti-CTLA-4 monotherapy is limited in humans (5) due to its relatively low response rate and high incidence of immune-related toxicity.

More recently, the combination of anti-PD-1/PD-L1 and anti-CTLA-4 antibodies has been tested to achieve better clinical benefits compared to each monotherapy. In patients with melanoma, combination blockade using nivolumab (anti-PD-1) and ipilimumab (anti-CTLA-4) improved the objective response rate (ORR), progression-free survival (PFS), and overall survival (OS) (12–14), as compared to ipilimumab alone. Similarly, numerically better ORR was reported with combination therapy in patients with non-small cell lung cancer compared to nivolumab monotherapy (15). Because distinct mechanisms of action have been suggested for anti-PD-1/PD-L1 and anti-CTLA-4 therapies (5), the combination approach is considered promising for overcoming resistance to each monotherapy. In addition to immunotherapy-naïve patients, the clinical benefits of combination therapy have been explored in patients who were refractory to anti-PD-1/PD-L1 monotherapy. In patients with melanoma who experienced disease progression following prior anti-PD-1/PD-L1 therapy (including innate and acquired resistance), significant antitumor activity was observed with combination therapy using nivolumab or pembrolizumab (anti-PD-1) plus ipilimumab (16, 17). These findings demonstrate the potential of the combination approach as a salvage treatment following the failure of anti-PD-1/PD-L1 monotherapy.

Cancers in dogs are gaining attention as comparative models for human cancer, leveraging the fact that dog tumors arise spontaneously in immunocompetent settings, typically in older age, and exhibit key common molecular features such as gene mutations and signaling pathway alterations (18). Over the past decade, immune checkpoints have been extensively studied in canines, and several anti-PD-1/PD-L1 antibodies have been developed for therapeutic purposes (19–23). Veterinary clinical studies using these ICIs have demonstrated that their clinical efficacy and safety profiles are generally similar to those reported in humans (19, 20, 24–26), suggesting an overall similarity between canine and human antitumor immunity. However, the response rate to anti-PD-1/PD-L1 monotherapy remains low in dogs, emphasizing the need for effective combination therapies. Anti-CTLA-4 antibodies have also been developed and characterized *in vitro* for canine cancer treatment (27, 28); however, no clinical studies have been conducted to date to evaluate their clinical efficacy and safety. To achieve greater clinical benefits for dogs with tumors and to enhance the value of canine cancers as comparative models for human cancer research, the development of a clinically relevant anti-CTLA-4 antibody is urgently required.

To address this, in this study, we demonstrated that the immunosuppressive role of CTLA-4 is conserved in the canine immune system by confirming that canine CTLA-4 competes with CD28 for ligands and downregulates cytokine production in canine immune cell cultures. Next, anti-CTLA-4 monoclonal antibodies were newly established and characterized for their binding properties and functional blockade of the CTLA-4 axis. A caninized (canine-ized) anti-CTLA-4 antibody, ca1C5, was then developed for therapeutic purposes, and its overall safety and pharmacokinetics were evaluated in a laboratory dog. Finally, the clinical efficacy and safety of ca1C5 in combination with the anti-PD-L1 antibody (c4G12) were explored in a pilot veterinary clinical study involving 12 dogs with advanced tumors refractory to prior c4G12 monotherapy.

## Results

### CTLA-4 impedes immune cell activation via ligand competition in dogs

**Figure 1:**
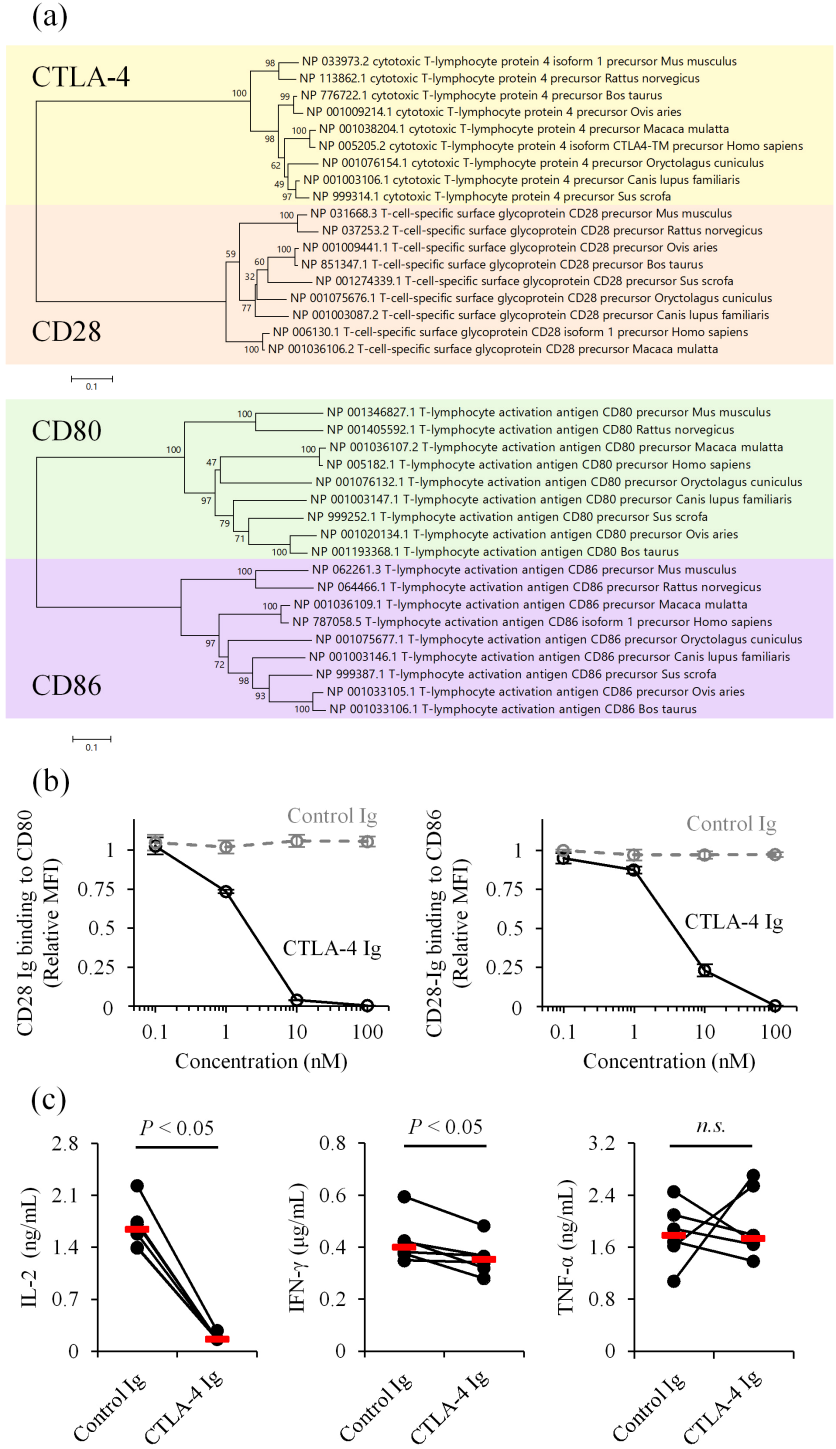
Inhibition of CD28 costimulation by canine CTLA-4. **(a)** Phylogenetic trees of mammalian CTLA-4, CD28, CD80, and CD86. Bootstrap percentages from 1,000 replicates are shown next to the branches. The scale bar indicates evolutionary distances (number of amino acid substitutions per site). **(b)** Competition of CD28 ligands by canine CTLA-4. CD28 Ig binding (relative mean fluorescence intensity, MFI) to CD80- or CD86-expressing cells was assessed in the presence of canine CTLA-4 Ig by flow cytometry. Dog IgG was used as a control (Control Ig). Data are presented as the mean of three independent experiments, with error bars indicating SEM. **(c)** Suppression of immune cell activation by canine CTLA-4. PBMCs from healthy dogs (*n* = 6) were cultured with superantigen for three days, and cytokine levels in the supernatant were measured by ELISA. Red bars indicate the median. Statistical analysis was performed using the Wilcoxon signed-rank test. *n.s.*, not significant.

To characterize the immunosuppressive role of CTLA-4 in canine immune responses, we first compared deduced amino acid sequences of mammalian *CTLA-4*, *CD28*, *CD80*, and *CD86* reported in the GenBank database. The sequence identity between human and canine CTLA-4 at the amino acid level was 87.4%, which was higher than the identity between human and mouse CTLA-4 (74.9%). Similarly, the sequence identities of CD28, CD80, and CD86 were 80.5%, 53.9%, and 62.6%, respectively, between humans and dogs, in contrast to 69.4%, 46.0%, and 58.1% between humans and mice. Phylogenetic analysis confirmed that canine orthologs are more closely related to their human counterparts than to mouse orthologs (**Figure 1a**). Notably, the B7-binding motif in the extracellular IgV domain and the cytoplasmic tail are 100% conserved among human, dog, and mouse CTLA-4 (**Supplementary Figure 1**). These results suggest that dogs may share a common immune regulatory pathway via CTLA-4 that is evolutionarily more relevant to the human immune system.

Next, we prepared recombinant proteins of canine CD28 and CTLA-4 to test ligand competition in a cell-based assay. The recombinant CD28 and CTLA-4 were expressed and purified in a soluble form, with the extracellular region fused to an IgG Fc region as a tag (CD28 Ig and CTLA-4 Ig). Binding of canine CD28 Ig to canine CD80- or CD86-expressing cells was detected using flow cytometry in the presence of various concentrations of canine CTLA-4 Ig. The addition of CTLA-4 Ig reduced CD28 Ig binding to both CD80- and CD86-expressing cells (**Figure 1b**). Consistent with this finding, treatment with CTLA-4 Ig in canine peripheral blood mononuclear cell (PBMC) cultures stimulated with a superantigen, staphylococcal enterotoxin B, reduced IL-2 and IFN-γ concentrations in the culture supernatant (**Figure 1c**). These results suggest that canine CTLA-4 inhibits T-cell activation by decreasing availability of costimulatory ligands.

### Establishment of rat monoclonal antibody against canine CTLA-4

Several monoclonal antibody clones were established by immunizing rats with canine CTLA-4. Among these, two clones were selected for further characterization: 2G2-G8 for expression analysis and 1C5-E5 for ligand-binding inhibition. Surface plasmon resonance (SPR) analysis suggested that both 2G2-G8 and 1C5-E5 bind canine CTLA-4 with high affinity, exhibiting sub-nanomolar K_D_ values (2.31 ± 1.82 × 10^−10^ M and 2.63 ± 0.03 × 10^−10^ M, respectively), which are comparable to, or slightly better than, the K_D_ value of ipilimumab/human CTLA-4 binding (6.25 ± 0.87 × 10^−10^ M) (**Table 1**).

**Table 1:** Binding properties of anti-CTLA-4 antibodies to recombinant CTLA-4.

Analyte	Isotype	Ligand	k_a_ (×10^6^/Ms)	k_d_ (×10^−4^/s)	K_D_ (×10^−10^M)
2G2-G8	rat IgG_2a_, κ	caCTLA-4	0.41 ± 0.03	0.91 ± 0.65	2.31 ± 1.82
1C5-E5	rat IgG_2b_, κ	caCTLA-4	4.79 ± 0.01	12.60 ± 0.01	2.63 ± 0.03
ca1C5	canine IgG-B, κ	caCTLA-4	5.08 ± 0.10	14.20 ± 0.04	2.80 ± 0.03
Ipilimumab	human IgG1, κ	huCTLA-4	0.92 ± 0.01	5.74 ± 0.81	6.25 ± 0.87

The kinetic constants were determined by fitting data to the 1:1 kinetic binding model.

Data are presented as means ± SD from three independent experiments. k_a_, association rate constant; k_d_, dissociation rate constant; K_D_, equilibrium dissociation constant.

**Figure 2:**
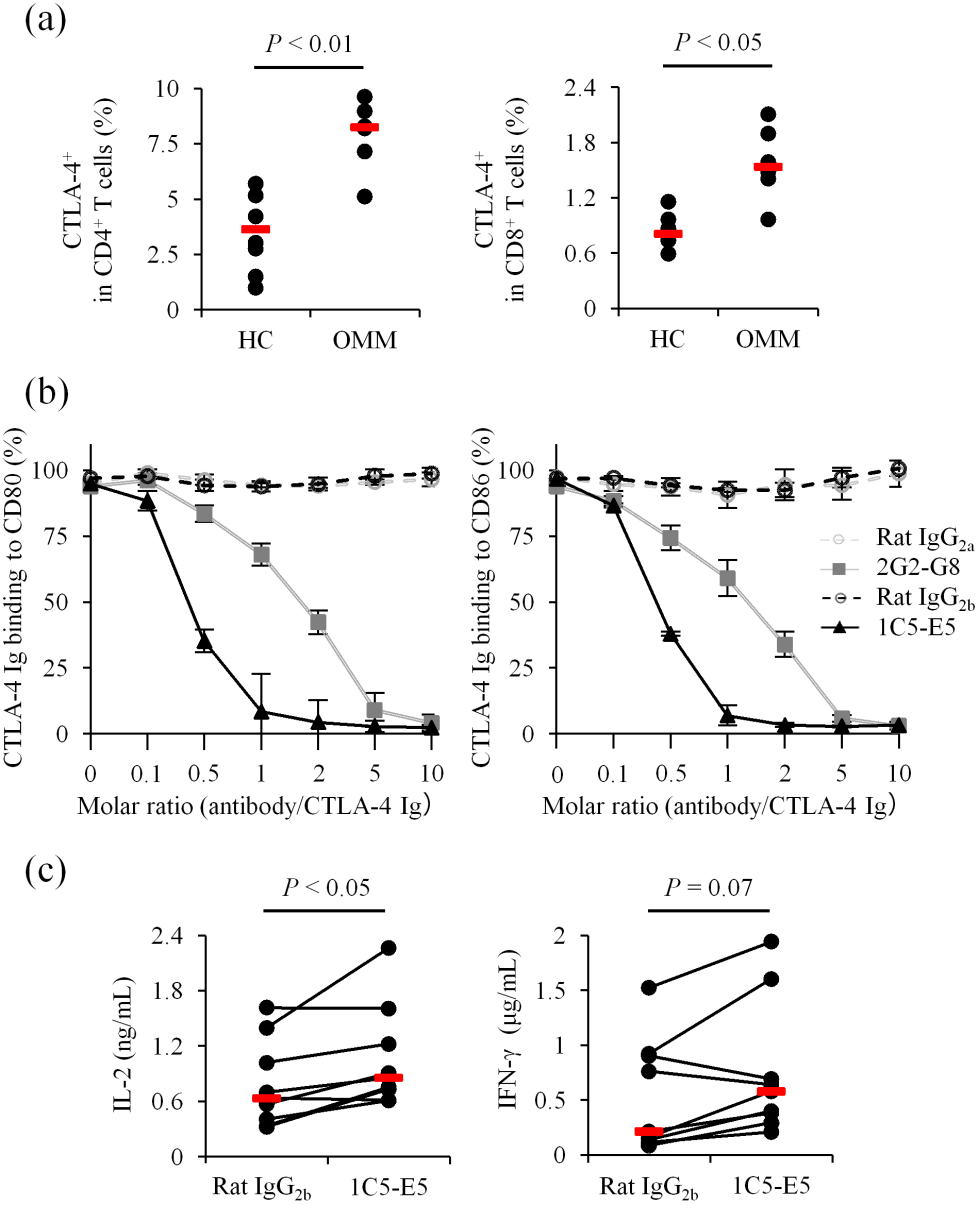
Detection and blockade of canine CTLA-4 using established monoclonal antibodies. **(a)** Flow cytometric detection of canine CTLA-4. WBCs from healthy dogs (*n* = 6) and OMM dogs (*n* = 6) were stained with 2G2-G8. Statistical analysis was performed using the Mann-Whitney U test. **(b)** Ligand binding blockade by anti-CTLA-4 monoclonal antibodies. CTLA-4 Ig binding (relative OD%) to CD80- or CD86-coated plates was assessed in the presence of each monoclonal antibody. Data are presented as the mean of three independent experiments, with error bars indicating SEM. **(c)** Enhancement of immune cell activation by 1C5-E5. PBMCs from healthy dogs (*n* = 9) were cultured with superantigen for three days and cytokine levels in the supernatant were measured by ELISA. 1C5-E5 was used at 10 μg/mL. Red bars indicate the median. Statistical analysis was performed using the Wilcoxon signed-rank test.

In flow cytometric analysis, 2G2-G8 detected endogenous levels of CTLA-4 expressed on peripheral blood T cells from healthy dogs (**Figure 2a**). Stimulation with a superantigen increased CTLA-4 expression on T cells in PBMC cultures (**Supplementary Figure 2**, same experimental condition as **Figure 1c**), suggesting the formation of a negative feedback loop upon T-cell activation. Higher CTLA-4 expression was observed on both CD4^+^ and CD8^+^ T cells in peripheral blood from dogs with oral malignant melanoma (OMM) (**Figure 2a**), implying that the CTLA-4 axis is a potential target for therapeutic intervention in canine cancer immunotherapy.

We next tested whether the established monoclonal antibodies inhibit the binding of CTLA-4 to its ligands, CD80 and CD86. A recombinant protein-based assay was performed using CD80 Ig- or CD86 Ig-coated microwell plates to detect CTLA-4 Ig binding to each ligand. Preincubation of CTLA-4 Ig with 2G2-G8 or 1C5-E5 reduced CTLA-4 Ig binding to the coated plates at higher antibody/CTLA-4 molar ratios (**Figure 2b**). Notably, 1C5-E5 achieved nearly complete inhibition at a lower molar ratio compared to 2G2-G8, supporting the use of 1C5-E5 for therapeutic purposes, specifically in T-cell activation. In canine PBMC cultures, treatment with 1C5-E5 increased IL-2 concentrations in the culture supernatant (**Figure 2c**), suggesting that 1C5-E5 inhibits CTLA-4 ligand binding and enhances T-cell activation through increasing the availability of costimulatory ligands for CD28 and/or by reducing the inhibitory signaling transmitted via CTLA-4.

### Characterization of caninized anti-CTLA-4 monoclonal antibody for canine cancer treatment

**Figure 3:**
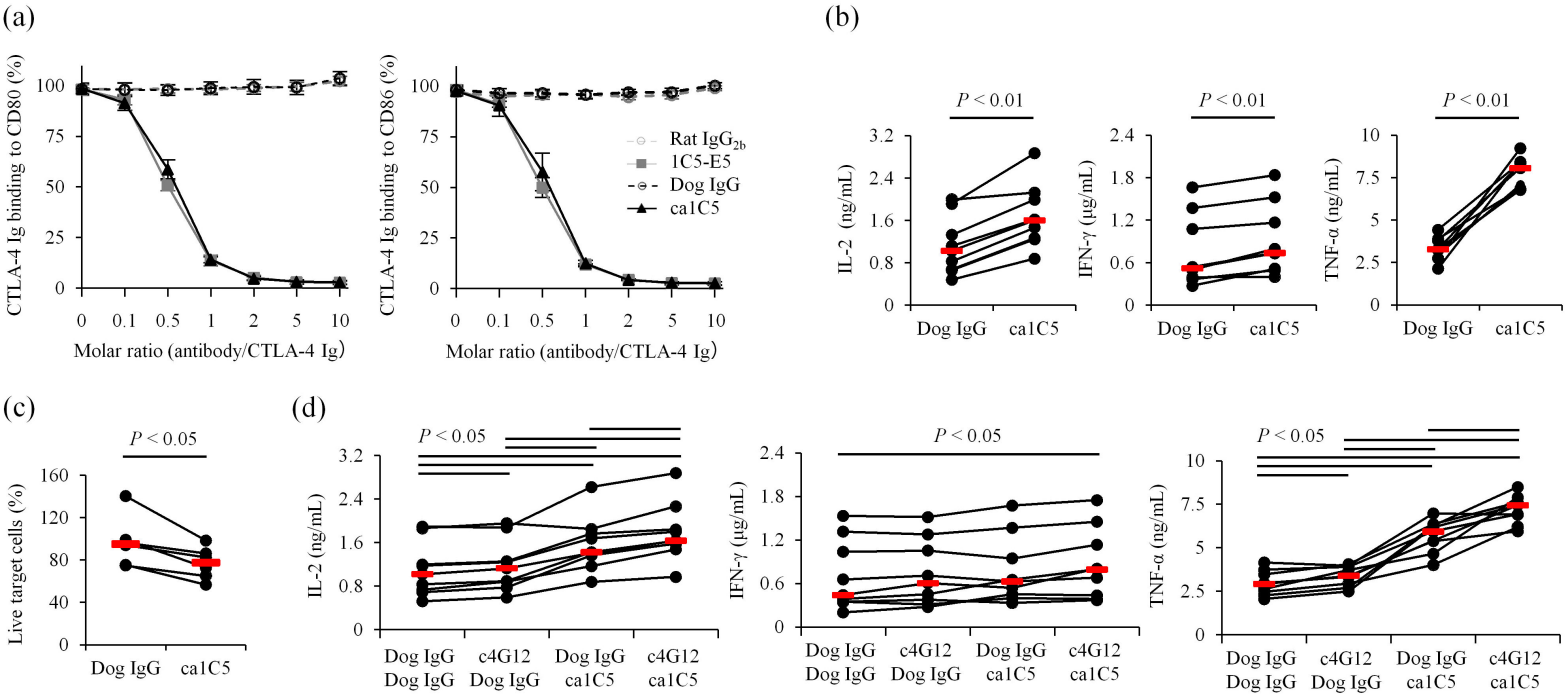
Characterization of caninized anti-CTLA-4 monoclonal antibody ca1C5. **(a)** Ligand binding blockade by ca1C5. CTLA-4 Ig binding (relative OD%) to CD80- or CD86-coated plates was assessed in the presence of each monoclonal antibody. Data are presented as the mean of three independent experiments, with error bars indicating SEM. **(b)** Enhancement of immune cell activation by ca1C5. PBMCs from healthy dogs (*n* = 9) were cultured with superantigen for three days and cytokine levels in the supernatant were measured by ELISA. **(c)** Induction of ADCC by ca1C5. IL-2–stimulated PBLs from healthy dogs (*n* = 6) were cocultured with CTLA-4–expressing target cells for 24 h in the presence of ca1C5. **(d)** Enhancement of immune cell activation by ca1C5 and c4G12. PBMCs from healthy dogs (*n* = 9) were cultured with superantigen for three days and cytokine levels in the supernatant were measured by ELISA. ca1C5 and c4G12 were used at 10 μg/mL. Red bars indicate the median. Statistical analysis was performed using the Wilcoxon signed-rank test with Holm’s correction for multiple comparisons.

To reduce the immunogenicity of the therapeutic antibody, the rat monoclonal antibody 1C5-E5 was converted into a canine IgG isotype by grafting the complementarity-determining regions (CDRs) into canine antibody frameworks. The resulting caninized antibody was named ca1C5, which retained binding properties almost identical to those of the original rat 1C5-E5 (**Table 1**). Indeed, a ligand-binding inhibition assay confirmed that ca1C5 was comparable to rat 1C5-E5 in blocking CTLA-4 Ig binding to CD80 Ig- or CD86 Ig-coated plates (**Figure 3a**). To further characterize its therapeutic potential, canine PBMC cultures were treated with ca1C5, and cytokine accumulation in the supernatant was measured as a surrogate indicator of T-cell activation. The concentrations of IL-2, IFN-γ, and TNF-α were significantly higher when ca1C5 was added to the culture (**Figure 3b**). Furthermore, the potential of ca1C5 to induce ADCC was evaluated in a cell-based assay using CTLA-4–expressing cells as target cells. The percentage of live target cells decreased in the presence of ca1C5 when these cells were cocultured with effector cells (IL-2–stimulated canine peripheral blood lymphocytes (PBLs)) (**Figure 3c**).

In clinical applications, ca1C5 is intended to be used in combination with anti-PD-1/PD-L1 antibodies, such as c4G12. The combination treatment with ca1C5 and c4G12 in canine PBMC cultures further increased IL-2 and TNF-α concentrations in the supernatant compared to ca1C5 or c4G12 treatment alone (**Figure 3d**), indicating an additive or synergistic stimulatory effect on T-cell activation. While IFN-γ concentrations were not significantly increased by either treatment alone, the combination treatment resulted in a statistically significant increase (**Figure 3d**).

### Safety and blood kinetics of anti-CTLA-4 antibody as monotherapy and in combination therapy with anti-PD-L1 antibody in a healthy dog

Before initiating a clinical study, we administered ca1C5 to a healthy laboratory dog to evaluate its safety and blood kinetics. The dosing regimen of ca1C5 was tentatively set at 1 mg/kg every 2 weeks, based on findings from human clinical studies using ipilimumab (9, 12–17). Repeated administration of ca1C5 (1 mg/kg) at 2-week intervals, for a total of four doses, did not induce any acute adverse events. The dog exhibited stable body temperature, pulse, and respiratory rate during and immediately after the infusions (**Supplementary Figure 3A**). Clinical evaluations, including physical examination, blood tests, urinary tests, and diagnostic imaging via X-ray and ultrasound, revealed no significant abnormalities (**Supplementary Figures 4, 5**). At 28 days following the fourth administration (day 70 from the first dose), a transient increase in the inflammatory marker C-reactive protein (CRP, 4.0 mg/dL) was observed. However, no clinical symptoms accompanied this finding, and the inflammatory site could not be identified. The CRP value returned to the normal range within one week without any intervention (0.6 mg/dL on day 77) (**Supplementary Figure 5**). To assess systemic immune activation, serum levels of cytokines, chemokines, and growth factors were measured using a multiplex immunoassay. All 11 factors tested showed no apparent increase after each ca1C5 administration (**Supplementary Figure 6**), suggesting the absence of nonspecific systemic immune activation, which might lead to immune-related adverse events. Notably, at the time of the transient CRP increase (day 70), elevated levels of IL-2, IL-6, IL-12, and SCF were detected. These elevations resolved by day 77, supporting the hypothesis that a mild, transient immune-related event occurred during this period.

After confirming the general safety of ca1C5 monotherapy, we proceeded to evaluate the safety of combination treatment using ca1C5 and c4G12. Starting on day 84, the dog received c4G12 (5 mg/kg), followed by a 30-min interval, after which ca1C5 (1 mg/kg) was infused. Repeated administration of both antibodies at 2-week intervals induced no acute adverse events (**Supplementary Figure 3B**). Interestingly, on day140 (14 days after the fourth combination treatment), a spike in CRP (7.3 mg/dL) was observed, although no clinical symptoms were present (**Supplementary Figure 5**). On the same day, mild pneumonia was detected via chest X-ray (asymptomatic and radiographic findings only; grade 1). This condition resolved without medical intervention by day143, coinciding with a decrease in CRP levels (0.8 mg/dL). To investigate reproducibility, a fifth combination administration was performed on day143. However, no CRP spike or radiographic changes were observed during the observation period (up to day 203). Throughout the study, the dog’s body weight increased slightly (**Supplementary Figure 4A**), suggesting no severe adverse events occurred during the experimental course.

**Figure 4:**
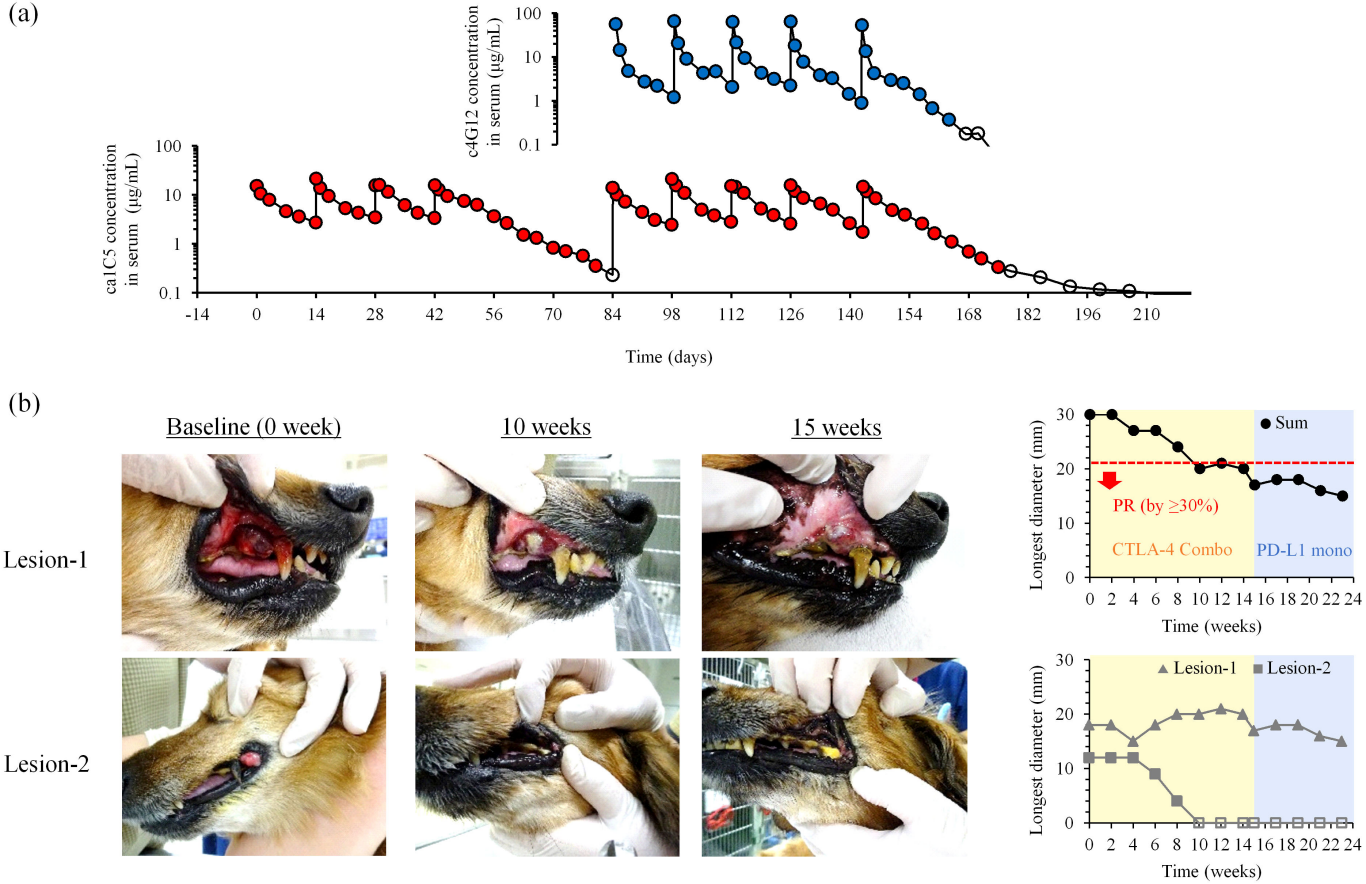
Blood kinetics and clinical efficacy of ca1C5 in combination with c4G12 immunotherapy. **(a)** Serum concentrations of ca1C5 and c4G12 during ca1C5 monotreatment and combination treatment. A laboratory Beagle received four doses of ca1C5 (1 mg/kg) every 2 weeks, followed by five doses of ca1C5 (1 mg/kg) and c4G12 (5 mg/kg). Open circles indicate concentrations below the lower limit of quantification (< 0.3 μg/mL). **(b)** Tumor response to combination therapy after the failure of c4G12 monotherapy. Recurrent lesions in the oral cavity that had arisen during prior anti-PD-L1 therapy were monitored during and after combination therapy. PR, partial response. Open symbols indicate the disappearance of the lesion (0 mm).

Serum concentrations of ca1C5 increased immediately after infusion and declined gradually thereafter. During repeated administrations at 2-week intervals, similar kinetics were observed, with negligible drug accumulation in peripheral blood (**Figure 4a**). In combination with c4G12, serum ca1C5 levels followed a similar trend, with no apparent interference between the two antibody drugs. The observed serum concentrations of ca1C5 were consistent with the inhibitory concentration *in vitro* (**Figure 3a**), where the molar ratio of 10 (antibody concentration of 10 nM) corresponded to 1.5 μg/mL antibody.

Taken together, ca1C5 was well-tolerated at the tested dosage, both as monotherapy and in combination with c4G12, with minimal immune-related adverse events that were within the expected type and severity.

### Safety and clinical efficacy of anti-CTLA-4 antibody as a salvage combination therapy after the failure of anti-PD-L1 therapy in dogs with advanced tumors

To further evaluate the safety and explore the clinical efficacy of ca1C5, a clinical study was conducted at our veterinary teaching hospital involving 12 dogs with spontaneous tumors refractory to prior c4G12 therapy. The clinical study was planned as a proof-of-concept study to demonstrate the safety and provide evidence of antitumor activity of anti-CTLA-4 antibody using the predetermined dosing regimen. Ten dogs had malignant melanoma (7 oral, 2 digital, and 1 splenic), while the remaining two dogs had limb osteosarcoma and bladder transitional cell carcinoma. The study included various canine breeds, with a median baseline age of 13 years (range: 7–18 years). Among these dogs, one was intact male, eight were neutered males, and three were neutered females. All dogs had received at least one prior therapy (e.g., surgery, radiation, chemotherapy, and/or molecular targeted therapy) before undergoing anti-PD-L1 therapy with c4G12. Several dogs were treated with c4G12 as a maintenance therapy following radiation or as an adjuvant therapy after surgical resection of the tumor (**Table 2**, **Supplementary Table 2**). Despite continuous treatment, all dogs eventually developed progressive disease (PD) on c4G12 therapy, with a median PFS of 101.5 days (95% CI: 14–168 days) (**Supplementary Table 3**). After confirming tumor refractoriness to anti-PD-L1 therapy, salvage therapy with ca1C5 was initiated while continuing c4G12 at the same dose. At the baseline of the combination treatment, six dogs had at least one measurable lesion (“with target disease”) according to cRECIST (29), and the remaining six dogs had only non-measurable lesions (“with non-target disease”). The median number of ca1C5 combination treatments was four (range: 1–8), with a median treatment duration of 56 days (range: 1–112 days) (**Supplementary Table 4**). Nine dogs (75.0%) died or dropped out of the study due to disease progression. The remaining three dogs (25.0%) discontinued the combination therapy due to treatment-related adverse events (TRAEs).

**Table 2:** Characteristics of dogs (*n* = 12) at baseline of the combination therapy.

Characteristic	
Breed―no. (%)	
Airedale Terrier	1 (8.3)
American Cocker Spaniel	1 (8.3)
Chihuahua	1 (8.3)
Kaninchen Dachshund	1 (8.3)
Miniature Dachshund	3 (25.0)
Scottish Terrier	1 (8.3)
Siberian Husky	1 (8.3)
Mix	3 (25.0)
Age―years	
Median	13
Range	7–18
Sex―no. (%)	
Intact male	1 (8.3)
Neutered male	8 (66.7)
Intact female	0 (0)
Neutered female	3 (25.0)
Tumor type―no. (%)	
Malignant melanoma	
Oral	7 (58.3)
Digit	2 (16.7)
Spleen	1 (8.3)
Osteosarcoma	1 (8.3)
Transitional cell carcinoma	1 (8.3)
Prior therapy―no. (%)	
Surgery	8 (66.7)
Radiation	9 (75.0)
Cytotoxic chemotherapy	2 (16.7)
Molecular target therapy	2 (16.7)
Immunotherapy*	12 (100.0)
Measurable lesion―no. (%)	
Present	6 (50.0)
Absent	6 (50.0)

*All dogs were refractory to prior anti-PD-L1 immunotherapy at baseline.

TRAEs of any grade were reported in four dogs (33.3%), including grade 3 events in three dogs (25.0%). The most frequent TRAEs were elevated alkaline phosphatase (ALP), alanine aminotransferase (ALT), creatinine levels, and diarrhea, each observed in two dogs (16.7%) (**Table 3**). Notably, grade 3 elevations of ALT and ALP were observed in two dogs (Dog #4 and Dog #9), after the sixth and seventh combination doses, respectively, leading to treatment discontinuation. Both dogs subsequently returned to c4G12 monotherapy. At later time points, ALT and ALP levels showed a downward trend without further clinical intervention, suggesting that the liver toxicity was associated with CTLA-4 blockade. However, the exact cause of the enzyme elevations could not be identified. Another dog (Dog #5) experienced a grade 3 increase in creatinine after the second ca1C5 dose and discontinued the combination therapy after the third dose. Renal metastasis was identified in this dog at a later time point, making it unclear whether the renal dysfunction was directly induced by CTLA-4 blockade.

**Table 3:** Treatment-related adverse events (TRAEs) observed during the combination therapy (*n* = 12).

TRAEs―no. (%)	Any grade	Grade 3	Leading to discontinuation
Any TRAEs	4 (33.3)	3 (25.0)	3 (25.0)
ALP	2 (16.7)	2 (16.7)	2 (16.7)
ALT	2 (16.7)	2 (16.7)	2 (16.7)
Anorexia	1 (8.3)	0 (0)	0 (0)
Creatinine	2 (16.7)	1 (8.3)	1 (8.3)
Diarrhea	2 (16.7)	0 (0)	0 (0)
Vomiting	1 (8.3)	0 (0)	0 (0)

ALP, alkaline phosphatase; ALT, alanine aminotransferase.

Grading was performed according to the VCOG-CTCAE v1.1.

Among dogs with non-target disease (*n* = 6), one dog (Dog #3) died a day after the first combination treatment. The remaining five dogs experienced unequivocal disease progression within 8 weeks (**Supplementary Table 4**). Among dogs with target disease (*n* = 6), five dogs experienced PD as their best overall response. Notably, one dog with recurrent OMM (Dog #9) achieved a partial response (PR). Dog #9 initially presented with stage II OMM at our veterinary hospital and underwent hypofractionated radiation therapy (three 8 Gy fractions at 1-week intervals), achieving temporary local tumor control. Three months later, the oral tumor regrew, and four additional 8 Gy fractions of radiation therapy were administered, resulting in another tumor regression. One week after the last radiation dose, anti-PD-L1 therapy (c4G12, 5 mg/kg) was initiated as maintenance therapy to prevent tumor recurrence and metastasis. After 15 weeks of c4G12 therapy, local tumor recurrence was confirmed during a physical examination, revealing two measurable target lesions in the oral cavity (lesion-1 in the right maxillary gingiva and lesion-2 in the left lip commissure) (**Figure 4b**). On the same day, combination therapy with ca1C5 and c4G12 was initiated. The baseline tumor burden (30 mm) decreased by >30% (to 20 mm, PR) after 10 weeks of combination therapy. Lesion-1 exhibited significant changes in shape and color, with necrosis (white colored region) and some volume reduction but stable longest diameter throughout the observation period. Lesion-2 achieved complete remission by week 10, and this response was maintained until the end of the observation. After the seventh dose of ca1C5 (week 15), the combination therapy was discontinued due to liver toxicity and c4G12 monotherapy was resumed. The tumor burden continued to decrease gradually during monotherapy, suggesting that anti-PD-L1 monotherapy can serve as maintenance treatment following tumor reduction induced by combination therapy. The dog ultimately died of tumor-related complications (loss of appetite and gastric dilation) at week 24.

Collectively, treatment with ca1C5 in combination with c4G12 was well-tolerated in dogs with advanced malignant tumors, demonstrating its potential as a salvage therapy after the failure of c4G12 monotherapy.

## Discussion

In this study, the therapeutic potential of CTLA-4 blockade in canine cancers was investigated through the development of a caninized anti-CTLA-4 antibody, ca1C5, designed to block ligand binding to canine CTLA-4. The PBMC culture assays demonstrated the immunostimulatory effects of ca1C5, both as a single agent and in combination with the anti-PD-L1 antibody c4G12. Administration of ca1C5 to a healthy laboratory dog confirmed its overall safety and expected blood kinetics, whether used as monotherapy or in combination with c4G12, with minimal evidence of immune-related adverse events. Furthermore, the clinical study involving dogs with anti-PD-L1 refractory tumors showed that the combination treatment was generally tolerable. Among dogs treated with the combination therapy, one experienced a durable objective response that persisted even after discontinuation of ca1C5. These findings suggest that ca1C5 is a promising candidate for anti-CTLA-4 immunotherapy in dogs, particularly as a rescue combination therapy following the failure of anti-PD-L1 monotherapy.

The immunosuppressive effects of CTLA-4 expression are primarily attributed to ligand competition with the costimulatory receptor CD28 (5). Recombinant canine CTLA-4 (CTLA-4 Ig) effectively blocked CD28 binding to CD80 and CD86 in a cell-based assay and reduced cytokine production in canine PBMC cultures. These findings align with the high degree of conservation in amino acid sequences of these molecules among humans, mice, and dogs. Previous studies have shown that canine leukocyte/lymphocyte proliferation is suppressed by the addition of CTLA-4 Ig (30, 31), highlighting its potential application as an immunosuppressant for transplantation and autoimmune disease treatment. Indeed, human CTLA-4 Ig (abatacept) has been approved for treating adult rheumatoid arthritis and juvenile idiopathic arthritis. Conversely, peripheral blood T cells from dogs with OMM expressed CTLA-4 at higher levels than those from healthy dogs. This increased CTLA-4 expression in circulating T cells suggests a similar upregulation on tumor-infiltrating T cells, contributing to the formation of an immunosuppressive TME. The negative impact of CTLA-4 expression in the TME has been explored in canine tumor models. Previous studies revealed that CTLA-4 expression was detected via immunohistochemistry in tumor-infiltrating lymphocytes, and higher staining scores or frequencies of positive cells were associated with poor clinical outcomes in canine mammary gland and melanocytic tumors (32, 33). Extending such expression analyses to other tumor types could help identify dog populations most likely to benefit from anti-CTLA-4 therapy.

c4G12 (anti-PD-L1) monotherapy has previously been tested for its efficacy in dogs with various advanced tumors, including OMM, with reported ORRs of 7.7–25.0% (19, 24, 25). Most treated dogs exhibit primary resistance to anti-PD-L1 therapy, and the majority of responders eventually experience relapse or disease progression despite continued treatment (acquired resistance). Although the mechanisms underlying such resistance remain unclear, it is suggested that blockade of the PD-1/PD-L1 axis alone is insufficient to fully reverse the immunosuppressive TME in canines, similar to observations in humans. Ipilimumab has been used in combination with anti-PD-1 antibodies (nivolumab, pembrolizumab) in humans; however, as a fully human monoclonal antibody, it may be immunogenic to canines, and no cross-reactivity with canine CTLA-4 was observed in our SPR analysis. ca1C5 represents a clinically relevant anti-CTLA-4 antibody for therapeutic use in dogs because its immunogenicity is expected to be very low due to caninization, and its binding affinity to canine CTLA-4 is considered comparable to that of ipilimumab to human CTLA-4. Indeed, repeated administration to a healthy dog did not result in allergic reactions, suggesting its immunogenicity is within an acceptable range for canines. Moreover, evidence of clinical efficacy of ca1C5 was demonstrated in a dog with recurrent OMM. While the ORR should be calculated in further clinical studies involving a larger number of dogs with a uniform tumor type, it seemed to be modest. The low response rate is consistent with expectations given the anti-PD-L1 therapy–refractory nature of the tumors, and similar response rates (approximately 10–30%) have been reported in human clinical studies involving anti-CTLA-4 and anti-PD-1/PD-L1 combinations for refractory tumors (16, 17, 34–36). In contrast, significantly higher efficacy has been observed in immunotherapy-naïve patients, with ORRs of approximately 40–60% for combination therapy (12–15, 37, 38). This suggests that such therapeutic approaches could maximize clinical benefits for canine cancers and should be considered in future veterinary clinical studies. Because the ca1C5 clinical study used a fixed, predetermined dosing regimen, the optimal dose of ca1C5 in combination with c4G12 remains to be elucidated in future studies. A dose-escalation approach with careful monitoring of safety, antitumor efficacy, and target modulation in the treated animals (e.g., tumor-infiltrating T cell phenotyping) should provide useful information to identify the optimal dosing regimen of ca1C5. In addition, dogs with a variety of treatment histories were included in the clinical study. The impact of prior treatments other than c4G12 (e.g., surgery, radiation, and chemotherapy) should be considered in future studies as these may alter the immunologic tumor microenvironment and influence the treatment outcome.

While promising antitumor efficacy can be achieved through combination therapy, the frequency and severity of TRAEs may increase compared to monotherapy. In human clinical studies, grade 3 or higher TRAEs were reported in 30–60% of patients treated with nivolumab plus ipilimumab, with higher ipilimumab doses (3 mg/kg *vs*. 1 mg/kg) associated with an increased frequency of severe TRAEs (13, 15, 37, 38). Consistent with these findings, grade 3 TRAEs were observed in 25.0% (3 out of 12 dogs) in this study, including possible associations with liver and renal toxicities. Additionally, radiographic evidence of pneumonia was observed during the safety assessment, suggesting that excessive immune activation may affect various organs. Because toxicities involving the pulmonary, gastrointestinal, hematological, liver, endocrine, neurological, renal, dermatological, and pancreatic systems have been reported in human clinical studies (39), careful monitoring will be essential in future veterinary clinical studies using ca1C5 and c4G12. In addition, future studies should include the evaluation of anti-drug antibody (ADA) formation in the treated animals, which may affect the safety as well as the efficacy of the administered antibody drug.

The similar patterns of treatment response and safety profiles observed in anti-CTLA-4 antibody therapy for dogs with spontaneous cancers support the hypothesis that canine cancers are relevant models for human cancers, particularly in the context of ICI therapies. Dogs develop a wide range of tumors during their lifetimes, including some that are rare in humans (e.g., hemangiosarcoma, histiocytic sarcoma, osteosarcoma). Leveraging canine tumor models allows novel treatment strategies to be tested prior to human clinical trials, potentially reducing the likelihood of costly failures in human trials. Further studies are warranted to establish effective cancer immunotherapies for dogs and to better characterize canine ICIs as comparative translational models for human cancer research.

## Methods

### Animal samples

The use of animals in this study was approved by the Institutional Animal Care and Use Committee of Hokkaido University (#15–0149 and #20-0041). All experiments were conducted in accordance with the guidelines and regulations of the Faculty of Veterinary Medicine, Hokkaido University, which is fully accredited by the Association for Assessment and Accreditation of Laboratory Animal Care International. Peripheral blood samples were collected from clinically healthy, purpose-bred Beagle dogs aged 2 to 7 years. Additionally, samples were obtained from dogs with OMM treated at the Hokkaido University Veterinary Teaching Hospital (HUVTH).

### Cell preparations and cultures

PBMCs were isolated from heparinized peripheral blood using density-gradient centrifugation on Percoll (Cytiva, Tokyo, Japan). PBMCs were cultured in RPMI 1640 medium (Sigma-Aldrich, St. Louis, MO, USA) supplemented with 10% heat-inactivated fetal bovine serum (Thermo Fisher Scientific, Waltham, MA, USA), 100 U/mL penicillin, 100 μg/mL streptomycin, and 2 mM L-glutamine (Thermo Fisher Scientific) at 37°C with 5% CO_2_. White blood cells (WBCs) were prepared using Cell Lysis Solution (Promega, Madison, WI, USA), and were subjected to flow cytometric analyses to detect CTLA-4 on T cells. Chinese hamster ovary (CHO)-DG44 cells were cultured in CD DG44 medium (Thermo Fisher Scientific) supplemented with 20 mL/L of GlutaMAX (Thermo Fisher Scientific) and 18 mL/L of Pluronic F-68 (Thermo Fisher Scientific) at 37°C with 5% or 8% CO_2_. Expi293F cells were cultured in Expi293 Expression Medium (Thermo Fisher Scientific) at 37°C with 8% CO_2_.

### Sequence identity and phylogenetic analysis of canine *CTLA-4*

The deduced amino acid sequences of mammalian *CTLA-4*, *CD28*, *CD80*, and *CD86* (NCBI reference sequences) were retrieved from the GenBank database (https://www.ncbi.nlm.nih.gov). Sequence identity percentages at the amino acid level were calculated using the Protein BLAST program (https://blast.ncbi.nlm.nih.gov/Blast.cgi). Unrooted neighbor-joining trees were constructed using MEGA6 software (version 6.06) (40, 41) with default settings, except for 1,000 replicates for the bootstrap test.

### Preparation of enhanced green fluorescent protein-fusion protein expressing cells

To prepare canine CD80- or CD86-expressing cells, expression vectors encoding canine CD80 or CD86 fused to EGFP were constructed. Nucleotide sequences encoding canine CD80 (NM_001003147.1) or CD86 (NM_001003146.1) were amplified by PCR using gene-specific primers containing restriction enzyme cleavage sites (**Supplementary Table 1**) and inserted into the multicloning site of the pEGFP-N2 vector (Clontech, Palo Alto, CA, USA). The resulting plasmids were cloned and amplified in HST08 competent cells (Takara Bio, Shiga, Japan) and purified using FastGene Xpress Plasmid PLUS Kit (Nippon Genetics, Tokyo, Japan) or NucleoBond Xtra Midi Kit (Takara Bio). Expi293F cells (Thermo Fisher Scientific) were transfected with the plasmid using Expifectamine 293 transfection kit (Thermo Fisher Scientific) and cultured for two days prior to the ligand binding assay.

To prepare canine CTLA-4–expressing cells, expression vectors encoding canine CTLA-4 fused to EGFP were constructed. Nucleotide sequences encoding canine CTLA-4 (NM_001003106.1) were amplified by PCR using gene-specific primers containing restriction enzyme cleavage sites (**Supplementary Table 1**) and inserted into the multicloning site of the pEGFP-N2 vector (Clontech). The resulting plasmids were purified as described above. The expression vector was transfected into CHO-DG44 cells using Lipofectamine LTX (Thermo Fisher Scientific), and stably expressing cells were selected and cloned in supplemented CD DG44 medium containing 800 μg/mL G418 sulfate (Enzo Life Sciences, Farmingdale, NY, USA).

### Preparation of recombinant proteins

Canine CTLA-4 Ig (fused to canine IgG-D Fc) was prepared as a soluble protein, with the extracellular region of canine CTLA-4 (NM_001003106.1) fused to the Fc region of canine IgG-D (AF354267.1). The amino acid sequence of the fusion protein was designed and codon-optimized for expression in Chinese hamster (*Cricetulus griseus*) cells. The gene sequence was synthesized with AscI/ASiSI restriction sites (GenScript, Piscataway, NJ, USA) and inserted into the pDC62c5-U533 vector (42), and the plasmid was purified as previously described. CHO-DG44 cells (Thermo Fisher Scientific) were transfected with the plasmid using Lipofectamine LTX (Thermo Fisher Scientific), and stably expressing cells were selected and cloned in Opti-CHO medium supplemented with 4 mM GlutaMAX (Thermo Fisher Scientific). The stably expressing cell clone was cultured for 14 days in Dynamis medium (Thermo Fisher Scientific) supplemented with 4 mM GlutaMAX. The culture was fed with 3.3% v/v Efficient Feed B+ (3×) (Thermo Fisher Scientific) on days 3, 5, 7, and 10, and 4 g/L, 4 g/L, and 6 g/L glucose (Kanto Chemical, Tokyo, Japan; FUJIFILM Wako Pure Chemical, Osaka, Japan) on days 3, 5, and 7, respectively. CTLA-4 Ig was purified from the culture supernatant using Ab-Capcher ExTra (ProteNova, Kagawa, Japan) and the buffer was exchanged for PBS (FUJIFILM Wako Pure Chemical) using PD MidiTrap G25 (Cytiva). The protein concentration was measured using Pierce BCA Protein Assay Kit (Thermo Fisher Scientific).

Canine CTLA-4 Ig, CD28 Ig, CD80 Ig, and CD86 Ig (fused to rabbit IgG Fc) were prepared as soluble proteins using the extracellular regions of each molecule and the Fc region of rabbit IgG. Nucleotide sequences encoding the extracellular regions of canine CTLA-4 (NM_001003106.1), CD28 (NM_001003087.2), CD80 (NM_001003147.1), or CD86 (NM_001003146.1) were amplified by PCR using gene-specific primers with restriction enzyme cleavage sites (**Supplementary Table 1**) and inserted into the multicloning site of the pCXN2.1-Rabbit IgG Fc vector (a kind gift from Dr. Yokomizo, Juntendo University, Japan) (43, 44). The resulting plasmids were purified as described above, and the recombinant proteins were produced in Expi293F cells using ExpiFectamine 293 transfection kit (Thermo Fisher Scientific). Purification of the recombinant proteins was performed using Ab-Capcher ExTra (ProteNova). The buffer was exchanged for PBS (FUJIFILM Wako Pure Chemical) using PD MidiTrap G25 (Cytiva). Protein concentration was measured using Pierce BCA Protein Assay Kit (Thermo Fisher Scientific) or by ultraviolet (UV) absorbance at 280 nm with a NanoDrop 8000 Spectrophotometer (Thermo Fisher Scientific).

Recombinant canine CTLA-4 (CTLA-4–His) was prepared as a recombinant protein comprising the extracellular region of canine CTLA-4 tagged with a C-terminal 6× polyhistidine tag. The nucleotide sequence encoding the extracellular region of canine CTLA-4 (NM_001003106.1) was amplified by PCR using gene-specific primers with restriction enzyme cleavage sites (**Supplementary Table 1**) and inserted into the multicloning site of the pCXN2.1 vector (a kind gift from Dr. Yokomizo, Juntendo University, Japan) (44). The a polyhistidine tag sequence was added to the 3’ terminus of the amplicon using the reverse primer. The resulting plasmid was purified as described above, and CTLA-4–His was produced in Expi293F cells using ExpiFectamine 293 transfection kit (Thermo Fisher Scientific). Purification of the recombinant protein was performed using TALON Metal Affinity Resin (Takara Bio). The buffer was exchanged for PBS (FUJIFILM Wako Pure Chemical) using Amicon Ultra-15 Ultracel-3 (Merck Millipore, Burlington, MA, USA).

### Cell-based ligand competition assay

To evaluate ligand competition by CTLA-4, flow cytometric analysis was performed to detect CD28 Ig (fused to rabbit IgG Fc) binding to CD80- or CD86-expressing cells (EGFP-fusion, prepared as described above) in the presence of various concentrations of CTLA-4 Ig (fused to canine IgG-D Fc). Briefly, 2 × 10^5^ cells were incubated with 25 nM CD28 Ig, labeled with biotin using Lightning-Link Rapid Biotin Conjugation Kit (Type A) (Innova Biosciences, Cambridge, UK). CTLA-4 Ig was added at 0.1, 1, 10, and 100 nM. After washing, the cells were incubated with Streptavidin-APC (BioLegend, San Diego, CA, USA) and analyzed using a FACSVerse flow cytometer (BD Biosciences, San Jose, CA, USA). As a negative control for CTLA-4 Ig, dog IgG (Jackson ImmunoResearch, West Grove, PA, USA) was used at the same concentrations (Control Ig). In all steps, PBS containing 1% bovine serum albumin (Sigma-Aldrich) was used as the dilution and washing buffer. Data were presented as relative mean fluorescence intensities (MFIs), where the MFI (APC) of the test sample was divided by that of control cells stained only with CD28 Ig.

### PBMC cultures and quantification of cytokines in the supernatant

PBMCs from healthy dogs were cultured with 5 μg/mL Staphylococcal Enterotoxin B (Sigma-Aldrich) for three days. Canine CTLA-4 Ig (fused to canine IgG-D Fc), anti-CTLA-4 monoclonal antibody, or anti-PD-L1 monoclonal antibody c4G12 (19) was added at concentrations of 100 nM, 10 μg/mL, or 10 μg/mL, respectively. As negative controls, dog IgG (Jackson ImmunoResearch) or rat IgG_2b_ (LTF-2, Bio X Cell, Lebanon, NH, USA) was used at the same concentrations. Concentrations of IL-2, IFN-γ, and TNF-α in the culture supernatant were measured using Canine IL-2 DuoSet ELISA, Canine IFN-gamma DuoSet ELISA, and Canine TNF-alpha DuoSet ELISA kits (R&D Systems, Minneapolis, MN, USA), respectively.

### Anti-CTLA-4 monoclonal antibodies

Hybridomas producing rat anti-canine CTLA-4 monoclonal antibodies were established by immunizing rats with canine CTLA-4 (Cell Engineering Corporation, Osaka, Japan). A gene sequence encoding the extracellular region of canine CTLA-4 (NM_001003106.1) was cloned into a DNA immunization vector, and the plasmid was delivered to rats (WKY/Izm, 8-week-old) via electroporation. Lymphocytes were collected from immunized rats and fused with SP2 myeloma cells to generate hybridoma pools. Several cell clones producing monoclonal antibody (e.g., clones 1C5-E5 and 2G2-G8) were established using methylcellulose-based semi-solid medium or limiting dilution. The isotypes of the monoclonal antibodies were determined using Rat Immunoglobulin Isotyping ELISA Kit (BD Biosciences).

The caninized anti-CTLA-4 monoclonal antibody ca1C5 was generated by grafting CDRs of 1C5-E5 onto canine antibody frameworks. To identify the cDNA sequences encoding the heavy and light chain variable regions of 1C5-E5, total RNA was extracted from the hybridoma clone using TRIzol (Thermo Fisher Scientific), and gene fragments were amplified and sequenced using 5’-Rapid Amplification of cDNA Ends System Version 2.0 (Thermo Fisher Scientific). The amino acid sequences of the ca1C5 heavy and light chains were designed and codon-optimized for expression in CHO cells. The gene sequences were synthesized (GenScript) and inserted into the expression vector pDC62c5-U533 (42). The plasmid was transfected into CHO-DG44 cells using Lipofectamine LTX reagent (Thermo Fisher Scientific), and stable producer cell clones were established in Opti-CHO medium (Thermo Fisher Scientific) containing 4 mM GlutaMAX ((Thermo Fisher Scientific). The established producer cell clone was cultured for 14 days in Dynamis medium (Thermo Fisher Scientific) containing 4 mM GlutaMAX (Thermo Fisher Scientific). The culture was fed with 3.3% v/v Efficient Feed B+ (3×) (Thermo Fisher Scientific) on days 3, 5, 7, and 10, and with 4 g/L, 4 g/L, and 6 g/L glucose (FUJIFILM Wako Pure Chemical) on days 3, 5, and 7, respectively. ca1C5 was purified from the supernatant by affinity chromatography using MabSelect SuRe LX (Cytiva), and additional purification by anion-exchange chromatography using Q-Sepharose HP (Cytiva), followed by cation-exchange chromatography using CaptoSP ImpRes (Cytiva), was performed. Throughout the purification steps, HiScale 26/20 columns and an ÄKTA avant 150 chromatography system (Cytiva) were used. The buffer was exchanged for PBS using Vivaspin20 concentrators with 50 kDa molecular weight cut off membrane (Sartorius, Göttingen, Germany). The concentration of purified ca1C5 was measured by UV absorbance at 280 nm using a NanoDrop 8000 Spectrophotometer (Thermo Fisher Scientific).

### SPR analysis

The binding properties of monoclonal antibodies were evaluated via SPR analysis using canine CTLA-4–His (caCTLA-4–His) as the ligand. Anti-His tag antibody was immobilized onto a CM5 Sensor Chip (Cytiva) using His Capture Kit (Cytiva). CTLA-4–His was captured on the sensor chip, and each anti-CTLA-4 monoclonal antibody (at a maximum concentration of 20 nM) was applied as the analyte to detect binding and dissociation. Kinetic constants were determined through curve fitting using a 1:1 kinetic binding model. HBS-EP+ (Cytiva) was used as the dilution and running buffer. Similarly, kinetic constants between ipilimumab and human CTLA-4–His (huCTLA-4–His) were determined using InVivoSIM anti-human CTLA-4 (Ipilimumab Biosimilar) (Bio X Cell) and Human CTLA-4 His-tag Recombinant Protein (Thermo Fisher Scientific).

### Expression analysis of CTLA-4 on T cells of OMM dogs

Canine WBCs were incubated for 15 min in PBS containing 10% goat serum (Thermo Fisher Scientific) to prevent nonspecific antibody binding. The cells were then incubated with 2G2-G8 or rat IgG_2a_ isotype control (2A3, Bio X Cell), followed by another incubation with APC-conjugated goat anti-rat Ig secondary antibody (Southern Biotech, Birmingham, AL, USA). The cells were further stained with anti-dog CD3 FITC (CA17.2A12, Bio-Rad, Hercules, CA, USA), anti-dog CD8a PerCP-eFluor 710 (YCATE55.9, Thermo Fisher Scientific), and anti-dog CD4 (296712, R&D Systems), which was labeled with PE using Zenon Mouse IgG_2b_ Labeling Kit (Thermo Fisher Scientific). Stained cells were analyzed using a FACSVerse flow cytometer (BD Biosciences). Data were presented as percentages of 2G2-G8–bound cells (CTLA-4^+^ cells) within CD4^+^CD3^+^ lymphocytes (CD4^+^ T cells) or CD8^+^CD3^+^ lymphocytes (CD8^+^ T cells). The gating strategy is shown in **Supplementary Figure 7**.

### Recombinant protein-based inhibition assay for receptor-ligand binding

To evaluate the inhibition of receptor-ligand binding by anti-CTLA-4 antibodies, a colorimetric assay was performed to detect CTLA-4 Ig (fused to rabbit IgG Fc) binding to CD80 Ig- or CD86 Ig-coated microwell plates. Canine CD80 Ig or CD86 Ig (1 μg/mL) was coated onto MaxiSorp Immuno Plates (Thermo Fisher Scientific). Canine CTLA-4 Ig (1 nM), labeled with biotin using Lightning-Link Rapid Biotin Conjugation Kit (Type A) (Innova Biosciences), was preincubated with each anti-CTLA-4 monoclonal antibody at various antibody/CTLA-4 Ig molar ratios (0.1, 0.5, 1, 2, 5, or 10). Negative controls included the same concentrations of rat IgG_2a_, rat IgG_2b_ (Bio X Cell), and dog IgG (Jackson ImmunoResearch). The mixture was added onto the microwell plates, and CTLA-4 Ig binding was detected using NeutrAvidin HRP conjugate (Thermo Fisher Scientific) and TMB One Component Substrate (Bethyl Laboratories, Montgomery, TX, USA). The reaction was stopped with 0.18 M H_2_SO_4_, and absorbance at 450 nm was measured using an MTP-900 microplate reader (Corona Electric, Ibaraki, Japan). Data were presented as relative optical density (OD) values (%), calculated by dividing the OD of the test sample by that of control wells incubated without blocking antibody.

### ADCC assay

ADCC activity of ca1C5 was assessed in a cell-based assay using canine CTLA-4–EGFP-expressing cells as target cells and canine PBLs as effector cells. PBMCs from healthy dogs were cultured for 24 h with 200 ng/mL canine IL-2 (Kingfisher Biotech, Saint Paul, MN, USA). Non-adherent cells (PBLs) were collected and cocultured with CTLA-4–expressing cells for an additional 24 h at an effector-to-target ratio of 5:1. ca1C5 or dog IgG (Jackson ImmunoResearch) was added to the medium at 10 μg/mL. Cells were collected and incubated with Fixable Viability Dye (FVD) eFluor 780 (Thermo Fisher Scientific) and anti-CD14 (CAM36A, Washington State University Monoclonal Antibody Center, Pullman, WA, USA), which was labeled with PerCP-Cy5.5 using Lightning-Link Conjugation Kit (Innova Biosciences), to exclude dead cells and monocytes from the analysis, respectively. Live target cells (FVD^−^CD14^−^EGFP^+^ cells) were counted using CountBright Absolute Counting Beads (Thermo Fisher Scientific) and a FACSVerse flow cytometer (BD Biosciences). Data were presented as percentages of live target cells, calculated by dividing the absolute number of live target cells in the test sample by that in the control culture treated with PBS instead of antibody.

### Antibody administration and overview of the clinical study

A clinically healthy, purpose-bred Beagle (male, 2 years old) and tumor-bearing dogs (*n* = 12) presented at HUVTH received at least one dose of ca1C5. The clinical study was approved by the Institutional Animal Care Committee of Hokkaido University (#20–0041) and the Ethics Committee of the Faculty of Veterinary Medicine, Hokkaido University (#2022-001). The clinical study was planned to demonstrate the safety and to show evidence of antitumor activity of the combination treatment in dogs with advanced tumors refractory to prior c4G12 monotherapy. The inclusion criteria for study enrollment were as follows: (1) dogs with a histopathologic or cytopathologic diagnosis of malignant tumor, (2) dogs with clinically detectable tumors that can be monitored repeatedly during the study, (3) dogs with tumors that are not expected to be cured by existing therapies, and (4) dogs with written informed consent obtained from their owners. Given the exploratory nature of the clinical study, the presence of measurable lesions as defined by cRECIST (29) was not a prerequisite for enrollment. Dogs that met at least one of the following criteria were excluded from the study: (1) dogs with severe systemic illnesses unrelated to the tumor, (2) dogs with a history of severe immune-related disorders that may recur during the study, (3) dogs that were difficult to return for scheduled follow-up visits, or (4) dogs with extremely high body weight. The study period was from June 2022 to November 2024. ca1C5 was administered intravenously at a dose of 1 mg/kg every 2 weeks, infused over 30 min using a syringe pump. For combination treatments with c4G12, c4G12 was administered intravenously at a dose of 2 or 5 mg/kg over 1 h. After an interval of >15 min, ca1C5 was administered as described above. Premedication with diphenhydramine and famotidine was allowed in the clinical study.

To measure serum concentrations of the administered therapeutic antibodies, ELISAs were developed using CTLA-4 Ig (fused to rabbit IgG Fc) and PD-L1 Ig (45). MaxiSorp Immuno Plates (Thermo Fisher Scientific) were coated with 1 μg/mL CTLA-4 Ig or 10 μg/mL PD-L1 Ig and blocked with SuperBlock T20 (PBS) (Thermo Fisher Scientific). Serum samples were incubated, and bound antibodies were detected using HRP-conjugated anti-dog IgG2 antibody (for ca1C5, Bethyl Laboratories) or HRP-conjugated anti-dog IgG1 antibody (for c4G12, Bethyl Laboratories) and TMB One Component Substrate (Bethyl Laboratories). The reactions were stopped using 0.18 M H_2_SO_4_, and absorbance at 450 nm was measured using an MTP-900 microplate reader (Corona Electric).

### Safety assessment

Physical examinations and blood tests (complete blood count and blood biochemistry) were routinely conducted during treatment to monitor adverse events. Additionally, urinalysis, thoracic and abdominal radiography, and ultrasonography were performed when clinically indicated. Classification and grading of adverse events were based on the Veterinary Cooperative Oncology Group–Common Terminology Criteria for Adverse Events (VCOG-CTCAE) v1.1 (46). Serum levels of cytokines, chemokines, and growth factors, including IFN-γ, IL-10, IL-12/IL-23p40, IL-2, IL-6, IL-8, MCP-1, NGF-β, SCF, TNF-α, and VEGF-A, were measured using a bead-based multiplex immunoassay with Cytokine/Chemokine/Growth Factor 11-Plex Canine ProcartaPlex Panel 1 (Thermo Fisher Scientific) and the Luminex 200 System (Luminex, Austin, TX, USA). Data were presented as fluorescence intensity (FI) values.

### Evaluation of clinical efficacy

Tumor response was assessed using the response evaluation criteria for solid tumors in dogs (cRECIST) v1.0 (29). At baseline, six dogs had only non-measurable lesions as defined by cRECIST (i.e., <10 mm on CT/clinical examination or <20 mm on radiography/ultrasonography). Tumor response was classified as complete response (CR, disappearance of all detectable tumors), partial response (PR, ≥30% reduction in tumor burden), progressive disease (PD, ≥20% increase in tumor burden or the appearance of new lesions), and stable disease (SD, <30% reduction and <20% increase in tumor burden for at least six weeks). OS was defined as the time (days) from the first dose of the corresponding treatment to death, and PFS was defined as the time (days) from the first dose of the treatment to confirmation of PD or death.

### Statistical analyses

Statistical analyses were performed using EZR statistical software (version 1.35) (47), with *P* < 0.05 considered statistically significant. The Mann-Whitney U test was used for unpaired comparisons and the Wilcoxon signed-rank test was applied for pairwise comparisons. Holm’s *P* value adjustment method was used for multiple comparisons.

## Data Availability

The original contributions presented in the study are included in the article/**Supplementary Material**. Further inquiries can be directed to the corresponding author.
