# Supplementary material for: Development of caninized anti-CTLA-4 antibody as salvage combination therapy for anti-PD-L1 refractory tumors in dogs
Source: Front Immunol. 2025 May 20;16:1570717. doi: 10.3389/fimmu.2025.1570717 (PMC12130249; doi:10.3389/fimmu.2025.1570717)
Supplement: Supplementary file 1 [file DataSheet1.pdf]

Supplementary Materials for:

Development of caninized anti-CTLA-4 antibody as salvage combination therapy for anti-PD-L1 refractory tumors in dogs

Naoya Maekawa, Satoru Konnai*, *et al.*

*Corresponding author
email: konnai@vetmed.hokudai.ac.jp

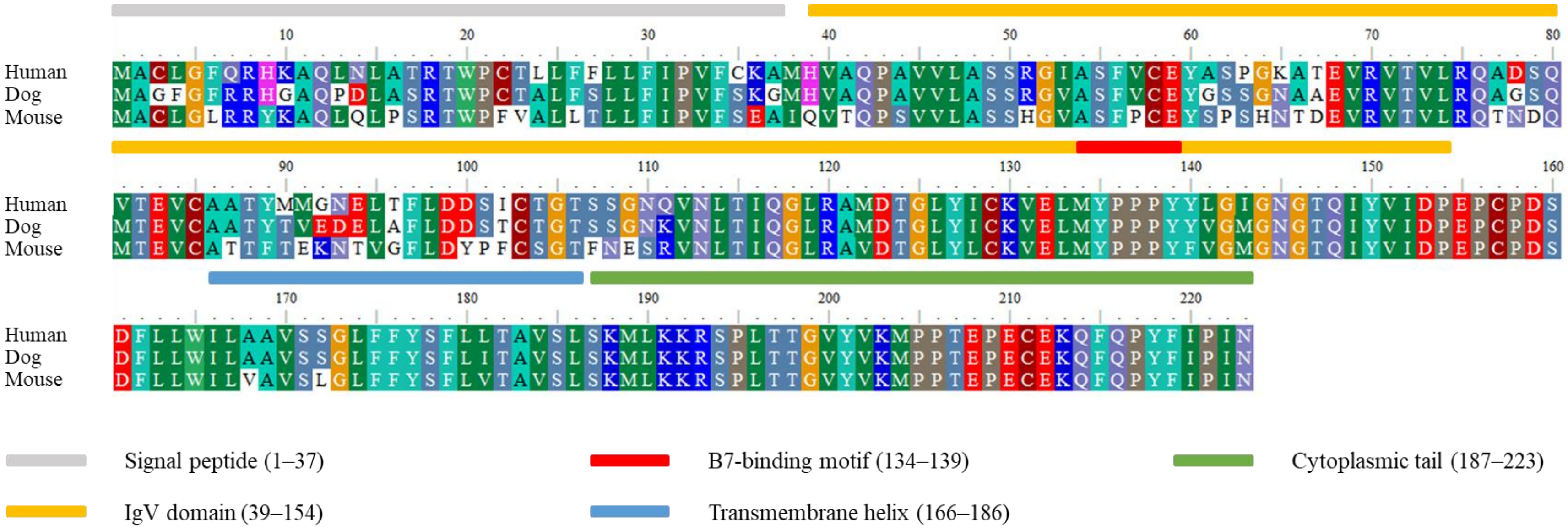

Supplementary Fig 1. Multiple sequence alignment of human, dog, and mouse CTLA-4.

Multiple alignment of CTLA-4 amino acid sequences in the human, dog, and mouse. The predicted signal peptide, immunoglobulin variable (IgV) domain, B7-binding motif, transmembrane helix, and cytoplasmic tail are indicated with numbers representing the positions of amino acid residues. Note that the B7-binding motif (MYPPPY) in the IgV domain and the cytoplasmic tail are 100% conserved among these animals.

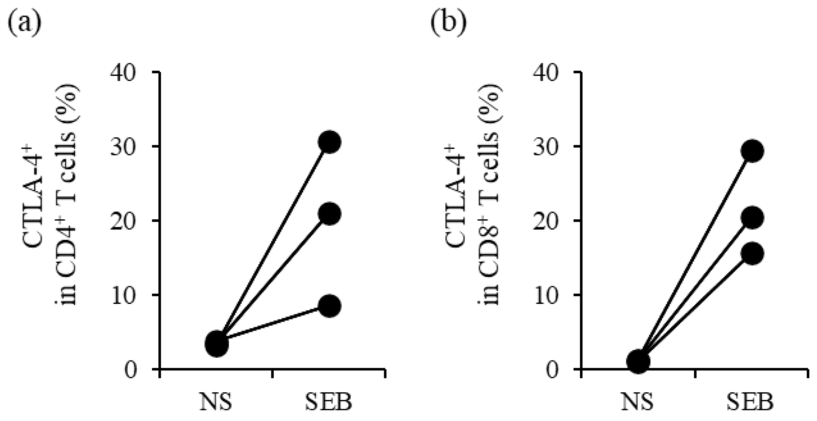

Supplementary Fig 2. CTLA-4 expression on T cells in stimulated PBMC cultures.
Canine peripheral blood mononuclear cells (PBMCs, *n* = 3) were cultured for 3 days in the presence of a superantigen, staphylococcal enterotoxin B (SEB). CTLA-4 expression was then analyzed on (a) $CD4^+$ or (b) $CD8^+$ T cells by flow cytometry. NS, no stimulation.

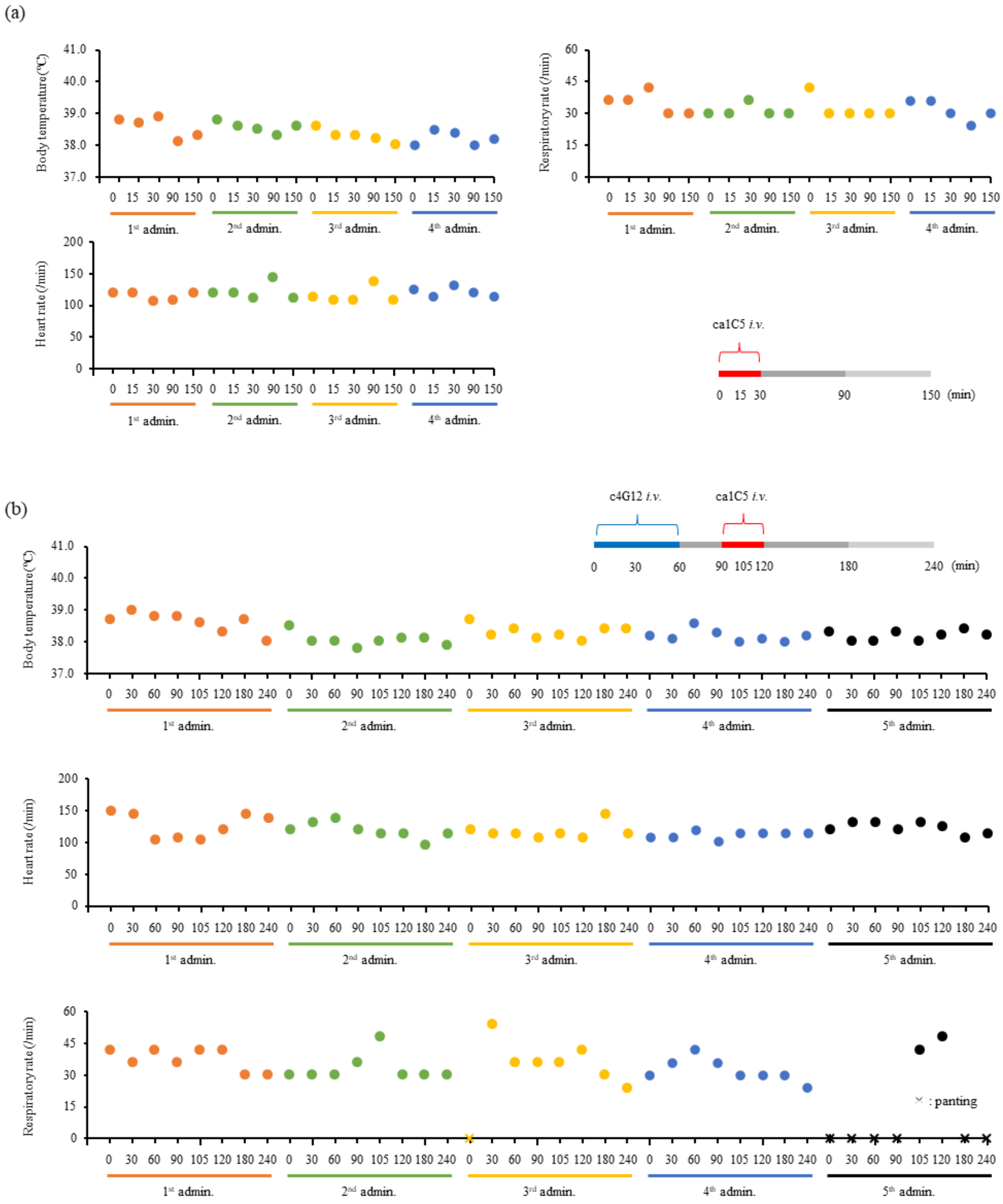

Supplementary Fig 3. Body temperature, pulse, and respiratory rate during and after the antibody administrations.

(a) Changes in vital signs during ca1C5 monotreatment. ca1C5 (1 mg/kg) was infused intravenously (*i.v.*) over 30 min, and the dog was monitored for an additional 2 h. (b) Changes in vital signs during combination treatment. c4G12 (5 mg/kg) was infused over 1 h, followed by a 30 min interval and infusion of ca1C5 (1 mg/kg) over 30 min. The dog was monitored for another 2 h post-administration.

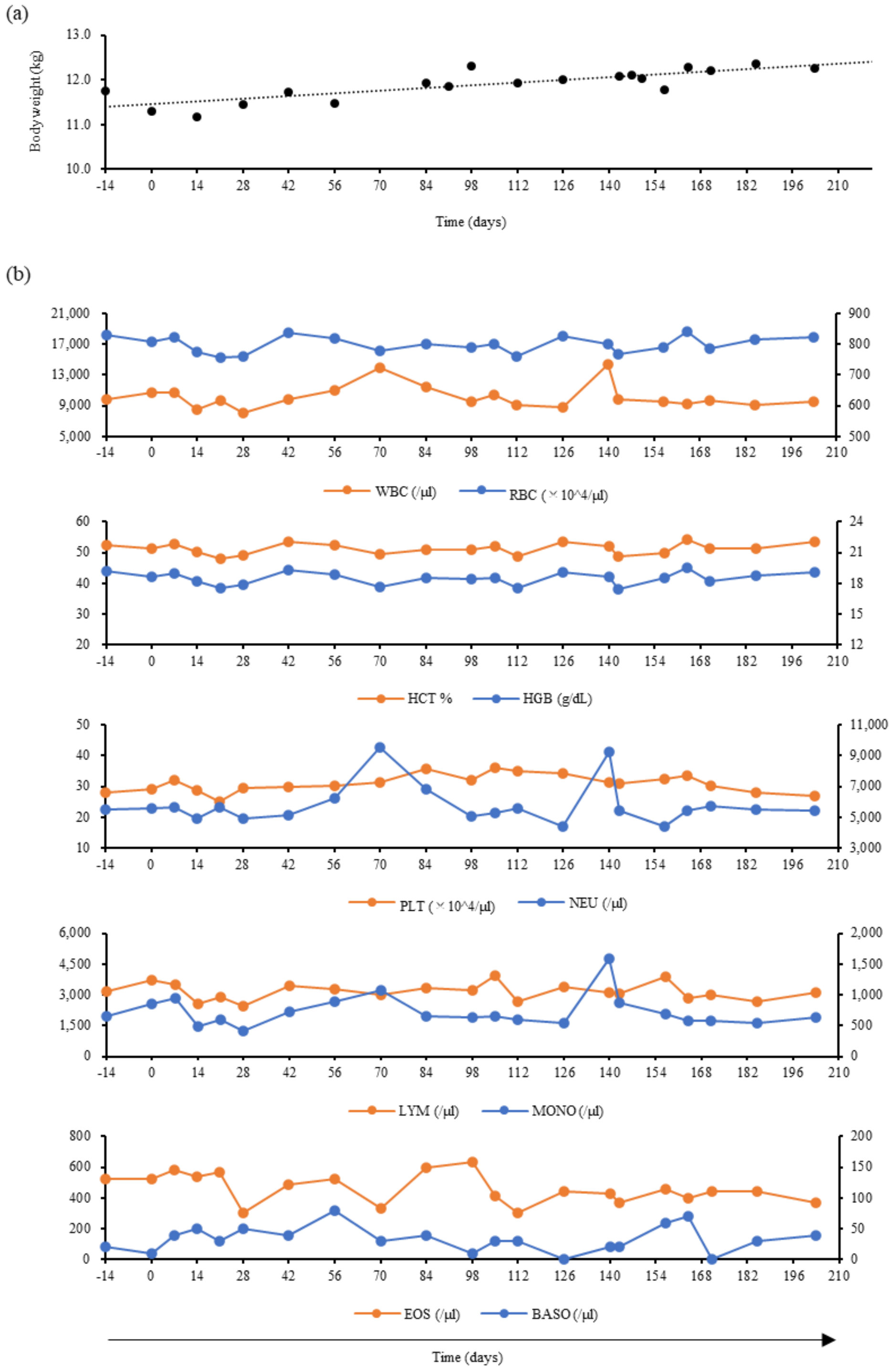

Supplementary Fig 4. Body weight and blood cell count.

(a) Changes in body weight during the study. The dotted line indicates a linear approximation. (b) Changes in blood cell count. Abbreviations: WBC, white blood cell; RBC, red blood cell; HCT, hematocrit; HGB, hemoglobin; PLT, platelet; NEU, neutrophil; LYM, lymphocyte; MONO, monocyte; EOS, eosinophil; BASO, basophil.

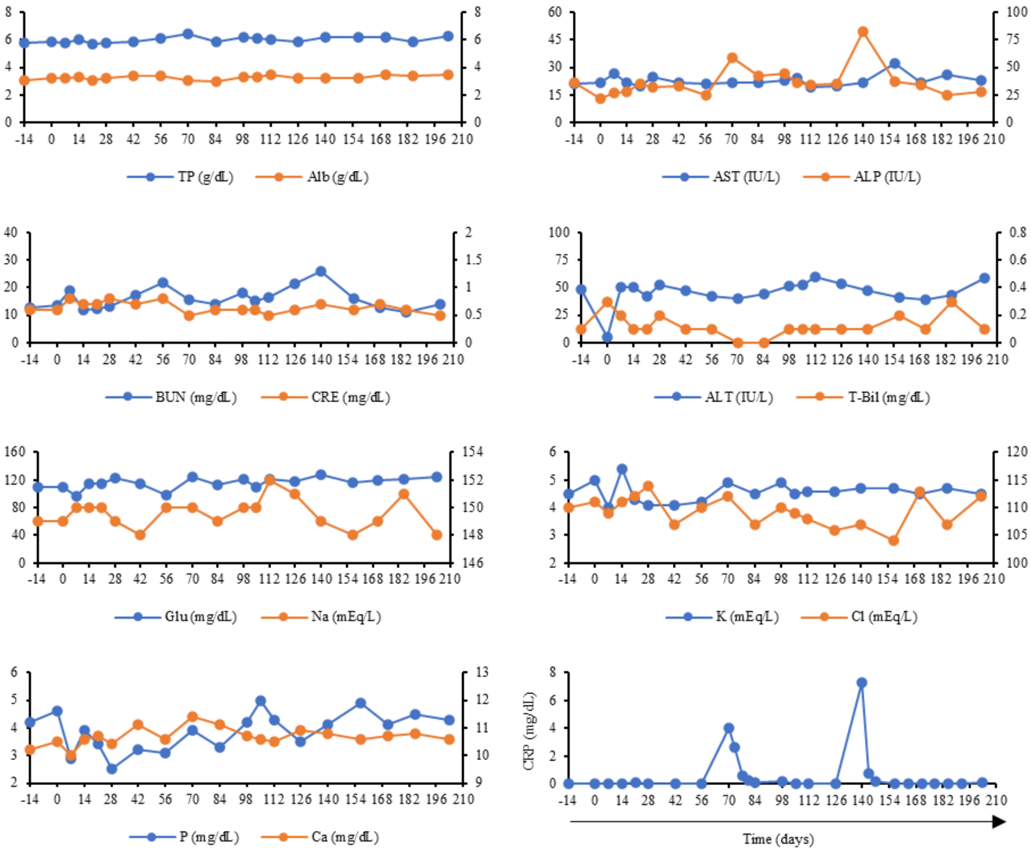

Supplementary Fig 5. Blood biochemistry.

Changes in blood biochemistry parameters during the study. Abbreviations: TP, total protein; Alb, albumin; AST, aspartate aminotransferase; ALP, alkaline phosphatase; BUN, blood urea nitrogen; CRE, creatinine; ALT, alanine aminotransferase; T-Bil, total bilirubin; Glu, glucose; CRP, C-reactive protein.

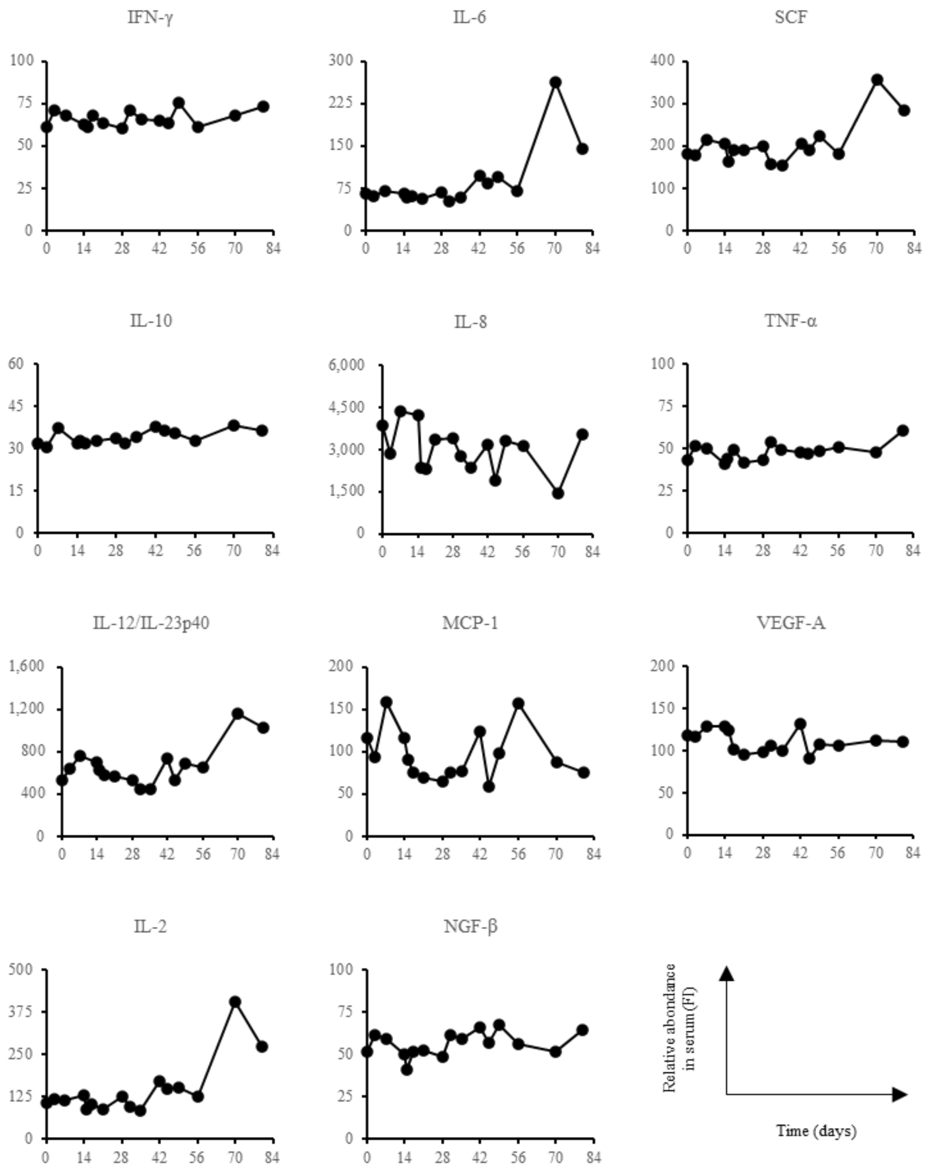

Supplementary Fig 6. Serum cytokine/chemokine/growth factors during ca1C5 monotreatment. Changes in serum levels of cytokines, chemokines, and growth factors during ca1C5 monotreatment. Relative abundance in serum is presented as fluorescence intensity (FI). Abbreviations: IFN, interferon; IL, interleukin; MCP, monocyte chemoattractant protein; NGF, nerve growth factor; SCF, stem cell factor; TNF, tumor necrosis factor; VEGF, vascular endothelial growth factor.

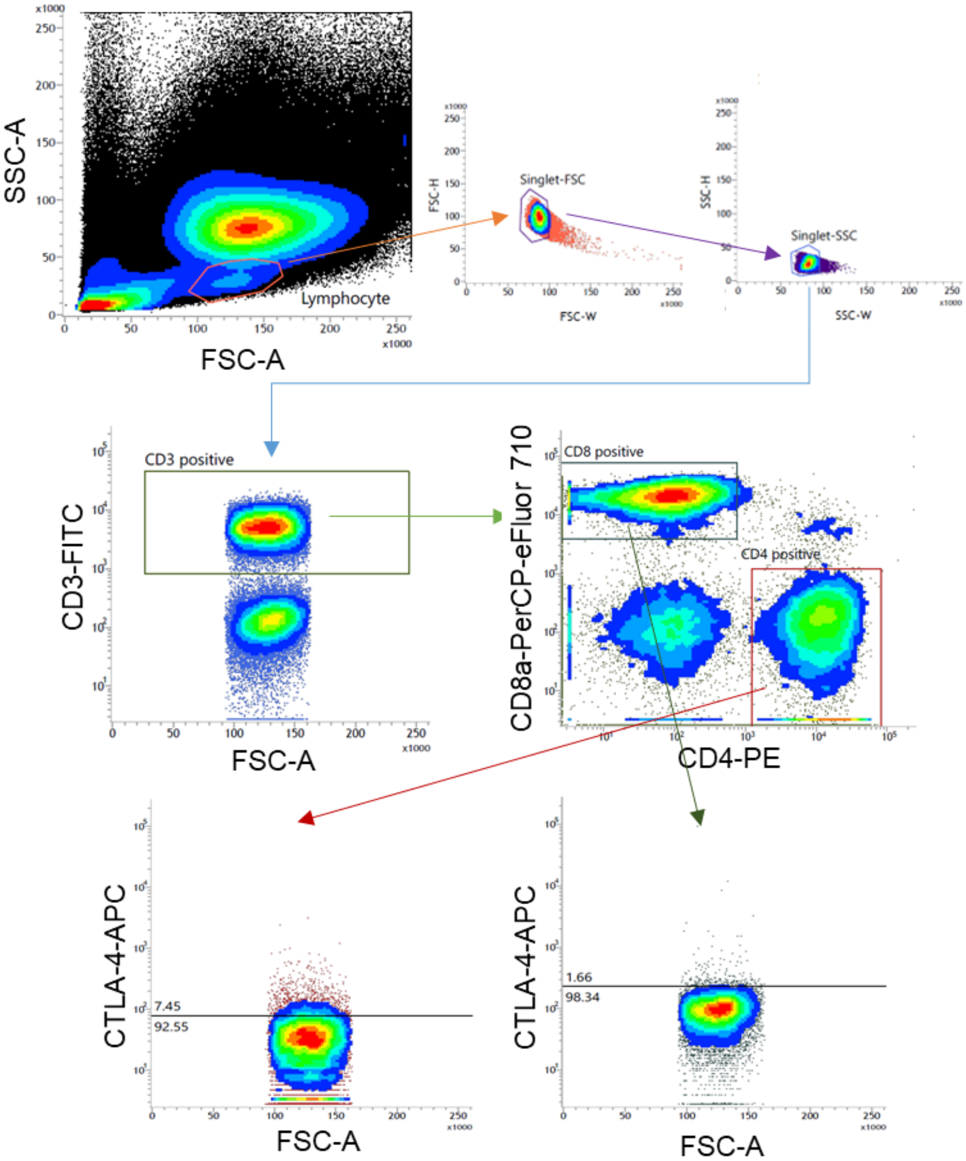

Supplementary Fig 7. Gating strategy and example plots for expression analysis of CTLA-4 on T cells.

CTLA-4 expression on $CD4^+$ or $CD8^+$ T cells ($CD3^+$ lymphocytes) was analyzed in white blood cells (WBCs) by flow cytometry.

Supplementary Table 1. PCR primers and restriction enzymes used in this study.

Primer name	Nucleotide sequence (5´–3´)	Restriction enzyme
CD80-EGFP_F	CGGGGTACCATGGATTACACAGCGAAGTG	KpnI
CD80-EGFP_R	TCCCCCGGGTACAGATGCTTGGGCAGATC	SmaI
CD86-EGFP_F	CCGCTCGAGATGTATCTCAGATGCACTA	XhoI
CD86-EGFP_R	TCCCCCGGGAAACTGTGTAGTACTGTT	SmaI
CTLA-4–EGFP_F	GAAGATCTATGGCTGGCTTTGGATTCCG	BglII
CTLA-4–EGFP_R	TCCCCCGGGATTGATGGGAATAAAATAAG	SmaI
CTLA-4 Ig_F	CGCGGATATCATGGCTGGCTTTGGATTCCG	EcoRV
CTLA-4 Ig_R	CGGGGTACCGTCAGAATCTGGGCAAGGTT	KpnI
CD28 Ig_F	CATGCTAGCATGATCCTCAGGCTGCTT	NheI
CD28 Ig_R	GGGGTACCCCAAAATGGCTTAGAAGA	KpnI
CD80 Ig_F	CGCGGATATCATGGATTACACAGCGAAGTG	EcoRV
CD80 Ig_R	CGGGGTACCCCAGAGCTGTTGCTGGTTAT	KpnI
CD86 Ig_F	CGCGGATATCATGTATCTCAGATGCACTAT	EcoRV
CD86 Ig_R	CGGGGTACCGTGGTCTCCATCAGGGGTG	KpnI
CTLA-4–His_F	CGCGGATATCATGGCTGGCTTTGGATTCCG	EcoRV
CTLA-4–His_R	CGGGGTACCTTAATGGTGATGGTGATGGTGGTCAGAATCTGGGCAAGGT	KpnI

Restriction enzyme recognition sites are underlined.

Supplementary Table 2. Characteristics of dogs treated with the combination therapy.

Dog #	Breed	Age (years)	Sex	Tumor type	Primary site	Metastatic site	Prior therapy
1	Mix	11	Female, spayed	Malignant melanoma	Oral cavity	LN, lung	Radiation, anti-PD-L1 (maintenance)
2	American Cocker Spaniel	15	Male, castrated	Malignant melanoma	Oral cavity	LN, lung	Surgery, radiation, anti-PD-L1
3	Chihuahua	12	Female, spayed	Malignant melanoma	Oral cavity	Lung, skin	Surgery, radiation, anti-PD-L1
4	Miniature Dachshund	18	Male, castrated	Malignant melanoma	Oral cavity	None	Radiation, anti-PD-L1
5	Airedale Terrier	10	Male, castrated	Malignant melanoma	Digit	Lung, skin	Surgery, anti-PD-L1
6	Mix	14	Male, castrated	Malignant melanoma	Oral cavity	None	Surgery, radiation, anti-PD-L1 (maintenance)
7	Mix	7	Male, castrated	Malignant melanoma	Spleen	Liver	Surgery, anti-PD-L1 (adjuvant)
8	Siberian Husky	11	Female, spayed	Osteosarcoma	Limb	Lung, bone	Surgery, cytotoxic chemotherapy, molecular targeted therapy, anti-PD-L1
9	Miniature Dachshund	17	Male, castrated	Malignant melanoma	Oral cavity	None	Radiation, anti-PD-L1 (maintenance)
10	Scottish Terrier	11	Male, intact	Malignant melanoma	Digit	LN, skin	Surgery, radiation, anti-PD-L1 (adjuvant)
11	Kaninchen Dachshund	15	Male, castrated	Transitional cell carcinoma	Bladder	None	Molecular targeted therapy, radiation, cytotoxic chemotherapy, anti-PD-L1
12	Miniature Dachshund	16	Male, castrated	Malignant melanoma	Oral cavity	Lung, skin	Surgery, radiation, anti-PD-L1

LN, lymph node.

Supplementary Table 3. Summary of prior anti-PD-L1 antibody monotherapy.

Dog #	PD-L1 expression	Dose	Number of doses	Treatment duration (days)	Measurable lesion	BOR	Concomitant therapy	PFS (days)	Remark
1	+	5 mg/kg	37	520	Absent	NA	None	240	
2	ND	5 mg/kg	5	70	Absent	NA	None	28	
3	+	5 mg/kg	1	14	Absent	NA	None	14	
4	ND	5 mg/kg	11	168	Absent	NA	Radiation	154	
5	+	2 mg/kg	21	300	Absent	NA	None	268	dog #2 in [25]
6	+	2 mg/kg	3	42	Absent	NA	None	42	
7	ND	5 mg/kg	14	196	Absent	NA	None	168	
8	+	2 mg/kg	10	147	Present	PR	None	119	dog #6 in [25]
9	+	5 mg/kg	7	126	Absent	NA	None	126	
10	ND	2 mg/kg	4	84	Absent	NA	None	84	
11	ND	5 mg/kg	3	49	Absent	NA	None	47	
12	ND	5 mg/kg	1	14	Absent	NA	None	14	

ND, not determined; BOR, best overall response; NA, not applicable; PR, partial response; PFS, progression-free survival.

Supplementary Table 4. Summary of anti-CTLA-4 antibody combination therapy.

Dog #	Number of doses	Treatment duration (days)	Measurable lesion	BOR	Concomitant therapy	PFS (days)	OS (days)	TRAEs (grade)
1	7	93	Present	PD	None	14	93	
2	2	28	Absent	NA	None	28	38*	Diarrhea (1)
3	1	1	Absent	NA	None	1	1	
4	6	84	Present	PD	None	28	181	Cre (1), ALT (3), ALP (3)
5	3	43	Absent	NA	None	15	134	Cre (3)
6	3	49	Present	PD	Surgery (day33)	28	49*	
7	8	112	Present	PD	None	28	112*	
8	4	56	Absent	NA	None	56	150	
9	7	105	Present	PR	None	165	165	Vomiting (1), ALT (3), ALP (3), Anorexia (1), Diarrhea (1)
10	4	56	Present	PD	None	28	114	
11	5	63	Absent	NA	None	56	146	
12	2	19	Absent	NA	None	14	19	

BOR, best overall response; PD, progressive disease; NA, not applicable; PR, partial response; PFS, progression-free survival; OS, overall survival; TRAEs, treatment-related adverse events; Cre, creatinine; ALT, alanine aminotransferase; ALP, alkaline phosphatase.
*Censored data.
